# USING A UNIVERSAL SATISFACTION SCORE IN LONG-TERM CARE SETTINGS

**DOI:** 10.1093/geroni/igz038.2726

**Published:** 2019-11-08

**Authors:** Nicholas Castle, Lindsay Schwartz, David Gifford

**Affiliations:** 1 WVU, Morgantown, United States; 2 American Health Care Association/National Center for Assisted Living, Washington, District of Columbia, United States

## Abstract

The CoreQ (not an acronym) consists of a limited number of satisfaction items (3-4 items, depending on setting) that are used to create an overall satisfaction score for long-term care facilities. This measure has been used in assisted living (AL) and skilled nursing facilities (SNFs) and has been endorsed by the National Quality Forum (NQF). Briefly, the development and psychometric testing of the CoreQ will be described, including the rationale for producing an overall satisfaction score and correlation with important quality indicators like Five-Star. Using data collected over the past 3 years, comprising more than 100,000 respondents, the use of the CoreQ measure will be described. For example, the CoreQ scores are used in MA to allow providers to benchmark their performance. The use of the scores in this way will be discussed including how providers have used the scores for quality improvement. Some states have elected to use CoreQ in pay for performance and other state initiatives. A case study of how New Jersey uses CoreQ with SNFs will be presented, including distribution of scores and addressing data collection challenges. CoreQ can be utilized as a short customer satisfaction measure to allow providers to benchmark their performance, residents and families in decision-making, and states and others to use for accountability.

